# Can probiotic-rich yogurt help prevent colorectal cancer in humans?

**DOI:** 10.1016/j.cpt.2025.06.002

**Published:** 2025-06-09

**Authors:** Biplab Adhikari

**Affiliations:** Department of Medicine, University of Louisville School of Medicine, 600 Marshall Street, Louisville, KY, 40202, USA

## Abstract

• Yogurt consumption is linked to 20–40% lower colorectal cancer risk, especially proximal colon tumors with high *Bifidobacterium* levels.
• Probiotics modulate gut microbiota, reduce inflammation, and produce protective metabolites against cancer development.
• *Bifidobacterium* strains in yogurt suppress tumor growth through various signaling and immune pathway regulation.
• Reducing the recommended age for early screening and increasing dietary fiber intake are recommended to enhance colorectal cancer prevention.
• Probiotic therapy shows promise for reducing treatment side effects and improving patient quality of life.

## Introduction

Colorectal cancer (CRC) remains a major global health challenge, ranking as the third most commonly diagnosed cancer and the second leading cause of cancer-related mortality worldwide.^1^ CRC develops when genetic mutations in kirsten-ras (*KRAS*), *TP53*, and adenomatous polyposis coli (*APC*), along with dysregulated signaling pathways such as wingless-type (Wnt)/β-catenin and transforming growth factor-β (TGF-β)/bone morphogenetic protein (BMP)/suppressor of mothers against decapentaplegic (SMAD), drive uncontrolled cell growth and impaired apoptosis in the colon or rectum, potentially leading to metastasis if left undetected.^1^

**Figure 1:**
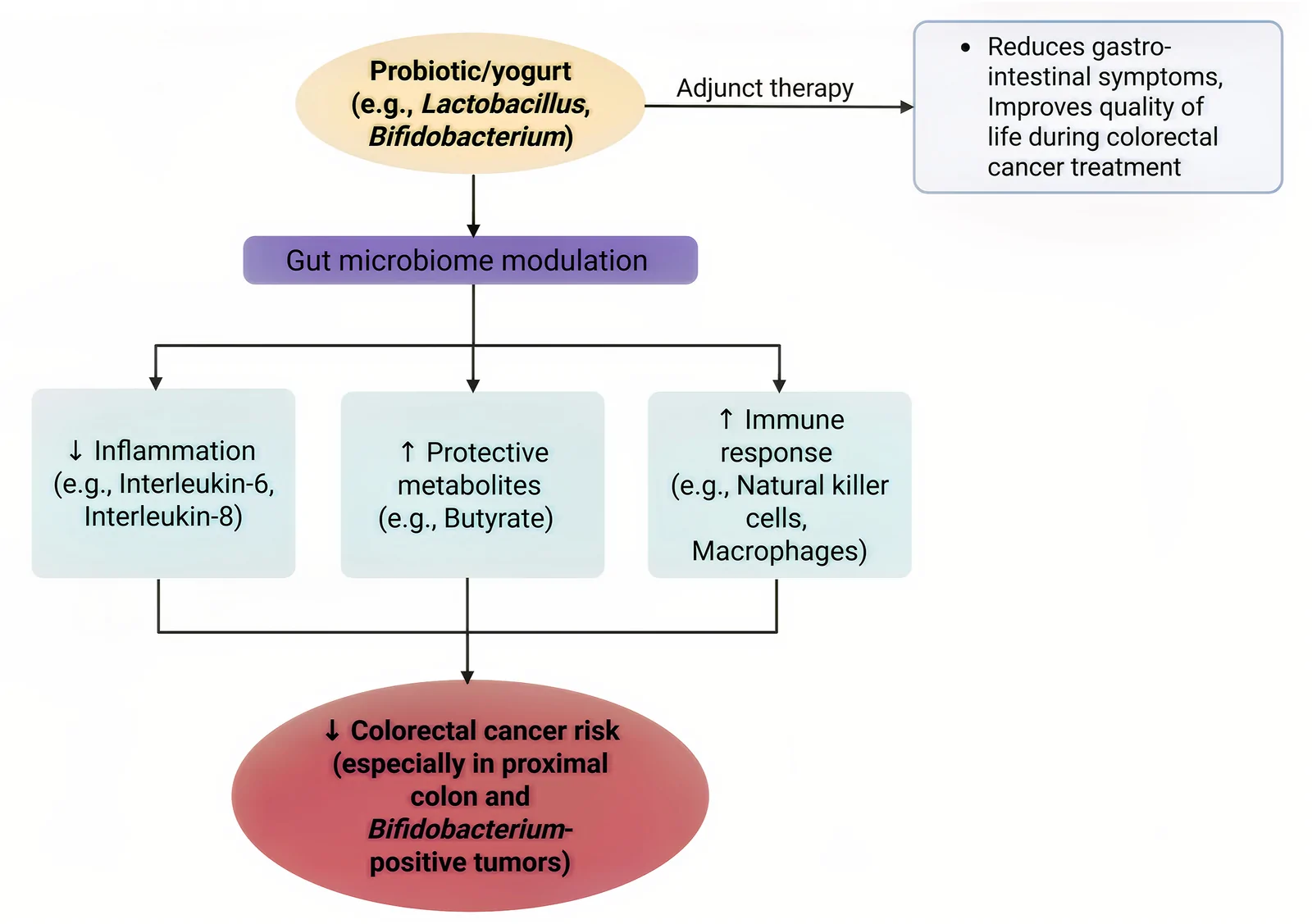
Summary of the protective roles of probiotic-rich yogurt in colorectal cancer prevention and adjunctive therapy.

While CRC has historically been predominant among individuals over age 50 years,^2^ recent data reveal a concerning rise in early-onset CRC, particularly among younger adults and certain ethnic groups, such as Hispanic populations.^1^^,^^3^ Evidence highlights the profound influence of diet, lifestyle, and the gut microbiome on CRC risk, positioning probiotic-rich yogurt as a promising adjunct in prevention efforts [Figure 1].^1^

## Epidemiology and risk factors

Multiple factors contribute to CRC, including genetic, environmental, and lifestyle factors. While age and family history remain critical non-modifiable risk factors, a significant portion of CRC risk is attributable to modifiable behaviors such as diet, physical activity, and substance use.^1^^,^^4^

### Diet and lifestyle

Recent studies highlight the role of diet and lifestyle in modifying gut microbiota, thereby influencing CRC development and progression.^1^^,^^4^^,^^5^ Factors such as obesity, physical inactivity, smoking, alcohol consumption, and environmental exposures can significantly disrupt microbial homeostasis, leading to dysbiosis and increased carcinogenic potential.^1^^,^^4^

Diets rich in fiber, polyphenols, and omega-3 fatty acids—abundant in plant-based foods and fermented products like yogurt—are associated with a more diverse and resilient gut microbiome.^1^ On the other hand, Western dietary patterns characterized by high intake of red and processed meats, saturated fats, and refined sugars contribute to the expansion of pro-inflammatory, tumorigenic bacteria.^1^^,^^5^ Physical activity, in contrast, supports microbial diversity and enhances mucosal immunity, offering protective effects against CRC.

Racial and ethnic disparities in CRC incidence and microbiome composition have been observed.^1^^,^^3^ For example, Hispanic individuals are more likely to be diagnosed with CRC at a younger age and tend to have a higher abundance of *Prevotellaceae*—a bacterial family implicated in both glucose metabolism and inflammation.^3^ Inter-individual variability in dietary exposures and microbial responses partially explains the heterogeneity in CRC outcomes across populations.^5^

### Molecular pathological epidemiology

Beyond lifestyle, genetic predispositions and epigenetic modifications also contribute to CRC pathogenesis. Inherited mutations, along with gut microbiota-induced changes in gene expression, play a central role in disease initiation and progression.^1^ Kwao-Zigah et al. emphasize that the gut microbiome is an active participant in modulating host gene regulation, rather than a passive inhabitant of the colon.^1^ Loss of beneficial commensals like *Bifidobacterium* and *Eubacterium* further disrupts mucosal immunity and gene regulation, reinforcing the link between gut dysbiosis and colorectal carcinogenesis.^1^ Furthermore, epigenetic mechanisms, such as DNA methylation and histone modification, can silence important tumor suppressor genes or activate oncogenes, setting the stage for cancer development.

Inamura et al. introduce the concept of the exposome, which encompasses all environmental exposures across the lifespan, as a framework for understanding how cumulative factors influence CRC risk.^6^ Diets high in animal fat and processed foods promote the proliferation of bacteria that produce carcinogenic metabolites and drive chronic inflammation, while diets rich in fruits, vegetables, and whole grains support protective microbial ecosystems.^1^^,^^6^ These exposures affect not only gut microbial composition but also systemic immune function, which may either suppress or permit tumor development.^6^

Hogue et al. report significant variability in gut microbiome composition across racial, ethnic, and socioeconomic groups.^3^ Such differences can affect susceptibility to CRC by altering the abundance of pro- or anti-inflammatory microbial populations. For instance, higher levels of inflammation-associated bacteria in certain populations may contribute to increased tumorigenesis.

Among microbial culprits*, Fusobacterium nucleatum* stands out due to its ability to modulate immune checkpoint pathways and promote immune evasion by tumor cells.^7^ As Luo et al. describe, *F. nucleatum* is linked to hypermethylation of critical genes such as MutL homolog 1 (MLH1) and Cyclin-dependent kinase inhibitor 2A (CDKN2A), which are involved in DNA repair and cell cycle control.^7^ Silencing of these genes facilitates genomic instability and uncontrolled cell proliferation.

Recent research also highlights the potential protective role of yogurt consumption, particularly formulations enriched with *Bifidobacterium.* According to Ugai et al., long-term yogurt intake increases the abundance of beneficial bacteria in the gut, which in turn produce short-chain fatty acids like butyrate.^8^ These compounds contribute to maintaining epithelial integrity, reducing oxidative stress, and modulating immune responses. On a molecular level, probiotics in yogurt inhibit genotoxic metabolite production and reduce DNA damage in colonic epithelial cells, supporting its role in CRC prevention.^8^

## Probiotics

Probiotics are live microorganisms that, when consumed in adequate amounts, provide health benefits primarily by modulating the gut microbiota. Yogurt, especially those with “live and active cultures” such as *Lactobacillus bulgaricus*, *Streptococcus thermophilus*, and *Bifidobacterium* species, is one of the most accessible sources of probiotics and has been studied for its roles in enhancing immune function, reducing inflammation, and potentially preventing CRC.^1^^,^^8^^,^^9^

Fermented foods such as kefir, kimchi, and sauerkraut may harbor a broader and more variable spectrum of microbes.^9^ These foods are supplemented with additional probiotic strains such as various *Lactobacillus* and *Bifidobacterium* species. Strain specificity, viability, and dosage play a crucial role in determining health outcomes.^1^ Synbiotics—combinations of probiotics and prebiotics may offer synergistic benefits by enhancing the growth and activity of beneficial bacteria. The distinction between probiotics in yogurt and those in other fermented products, such as cheese or kefir, lies in the type and viability of bacterial strains, their concentration, and their demonstrated health benefits.^10^

### Yogurt and CRC

A large prospective cohort study found that individuals who consumed at least one serving of yogurt per week had a significantly lower risk of developing proximal colon cancer [Table 1], compared with those who did not consume yogurt, with the protective effect being most notable when yogurt was consumed for a longer duration prior to diagnosis.^8^ Additionally, these findings were supported by other studies, reporting that yogurt intake was associated with a decreased risk of overall CRC across multiple populations and study designs.^11^ The proposed mechanisms include yogurt's positive influence on gut microbiota and its anti-inflammatory properties, though no significant association has been found between yogurt consumption and CRC mortality.^8^^,^^11^

**Table 1:** Summary of association between yogurt, cheese, and other fermented foods and colorectal cancer (CRC) risk.

Category	Key Findings	Reference(s)
Yogurt	Reduced risk of CRC, particularly in the colon and rectum. The strongest protective effect appears 16–20 years after consistent intake. This association may be influenced by probiotics and calcium in yogurt. No significant link has been found between yogurt intake and CRC mortality.	8,11
Cheese	Reduced risk of CRC, particularly for proximal colon cancer. The inverse relationship is clearer in case–control studies and European populations, though cohort study results are inconsistent. While cheese may offer protective effects, its high-fat content could potentially diminish these benefits.	12,13
Other fermented foods (non-dairy)	Demonstrate potential in suppressing CRC cell growth, reducing inflammatory cytokines, inhibiting cell proliferation, inducing cancer cell death, and disrupting antioxidant defenses. Protective effects may be mediated through modulation of gut microbiota and immune response. Most supporting evidence remains preclinical.	14

### Cheese and CRC

Cheese consumption has also been studied for its potential protective effects against CRC [Table 1]. Evidence suggests that higher cheese intake was associated with a lower risk of CRC, especially for proximal colon cancer.^12^ However, results across different studies are somewhat inconsistent; while some research indicates a significant inverse association, others have found no clear link between cheese or other fermented dairy products and CRC risk, except in certain populations such as those in Europe.^12^^,^^13^ The variability may be due to differences in cheese types, fermentation processes, and dietary patterns across regions. Overall, cheese appears to contribute to CRC risk reduction, but the evidence is less robust and more variable than for yogurt.

### Other non-dairy fermented foods and CRC

Beyond dairy products, other fermented foods have also attracted attention for their potential role in preventing CRC [Table 1]. Traditional fermented foods like Korean gochujang have shown anticancer effects *in vitro* by inhibiting CRC cell proliferation, inducing cell death, and disrupting cellular antioxidant defenses.^14^ While these findings are promising and suggest that fermented foods may exert protective effects through modulation of the gut microbiome and immune response, most evidence for non-dairy fermented foods is still preclinical, and more human studies are needed to confirm these benefits.^14^

## Mechanisms/evidence linking yogurt and CRC

The probiotic content of yogurt, such as *Lactobacillus* and *Bifidobacterium* species, collectively known as the gut microbiome can modulate the composition and have crucial roles in nutrient metabolism, immune regulation, and protection against pathogens in the colon.^8^^,^^10^ Disruptions to the gut microbiome composition and function, termed dysbiosis, have been associated with various pathological conditions, including CRC.^3^

### Lactococcus

A prominent probiotic bacterium found in yogurt and other fermented dairy products, produces nisin, a compound capable of suppressing the expression of various genes, proteins, and cytokines involved in cancer cell proliferation.^15^ These effects appear to be mediated through reduced cyclin D1 expression in SW480 cell lines, reduced natural killer (NK) cell activity, diminished cancer cell viability, and decreased levels of inflammatory cytokines including interleukin-8 (IL-8) and interleukin-6 (IL-6).^15^^,^^16^ Pre-administration of specific *Lactobacillus* strains can reduce tumor formation rates by over 86% and significantly suppress tumor growth through microbiota and metabolite modulation.^16^ These molecular changes collectively contribute to an environment less conducive to cancer cell growth and proliferation, potentially explaining yogurt's observed protective effects.

### Bifidobacterium

A growing body of epidemiological and experimental research supports an inverse association between yogurt consumption and CRC risk, particularly for *Bifidobacterium*-positive tumors.^8^^,^^17^ For instance, *Bifidobacterium adolescentis* has been found to modulate cancer-associated fibroblasts (CAFs) expressing angiotensin-converting enzyme (CD143^+^), thereby suppressing colorectal tumorigenesis through Wnt signaling-regulated Growth Arrest Specific 1 (GAS1) pathways.^17^

The most recent evidence from a 2025 study demonstrates a significant differential association between long-term yogurt consumption and CRC risk based on tumor *Bifidobacterium* abundance. Statistical analysis revealed a significant protective trend for *Bifidobacterium*-positive tumors among individuals consuming ≥2 servings/week of yogurt compared to those consuming <1 serving/month, while no such benefit was observed for *Bifidobacterium*-negative tumors.^8^ This differential pattern was particularly notable for proximal colon cancer, where yogurt consumption showed a stronger trend toward reduced incidence of *Bifidobacterium*-positive cases. Similar differential associations were not observed for distal colon or rectal cancers, suggesting anatomical specificity to this relationship.^8^

### Molecular effect

Molecular mechanisms underlying probiotic actions:

- Ingested probiotics compete with pathogenic bacteria for adhesion sites, helping to suppress harmful microbes and promote the production of anti-carcinogenic metabolites such as short-chain fatty acids like butyrate which inhibit tumor-promoting pathways and support healthy epigenetic regulation.^1^^,^^4^ They also bind and neutralize carcinogenic metabolites, such as secondary bile acids and lipopolysaccharides, thereby reducing epithelial proliferation and mutation rates implicated in CRC progression.^1^^,^^4^^,^^10^^,^^16^
- Yogurt-derived metabolites like d-lactate can activate specific receptors, such as Gi-coupled protein 81 (GPR81) to suppress pro-inflammatory macrophage polarization, facilitating mucosal repair and reducing tumorigenesis.^18^
- Probiotics have been shown to enhance natural killer cell activity and modulate the host immune response via macrophage activation and interleukin production, while their symbiotic association with prebiotics further boosts humoral immunity, collectively strengthening anti-tumor defenses.^10^

## Clinical applications

In treatment settings, scientific evidence suggests that probiotic supplementation can protect CRC patients from treatment-associated adverse effects, such as gastrointestinal symptoms and infections, while potentially improving their health-related quality of life (HRQoL) and postoperative recovery.^4^^,^^19^ Probiotics may reduce the incidence of diarrhea and infectious complications, improve bowel function and gut barrier integrity, and attenuate postoperative inflammatory responses in CRC patients receiving chemotherapy, radiotherapy, or surgical interventions.^4^^,^^19^

However, while these short-term clinical benefits are promising, more research is needed to determine whether probiotics can improve long-term outcomes such as progression-free survival and overall survival in CRC patients.^19^ The beneficial impact of probiotic supplementation appears to depend on factors such as the specific strains used, dosage, duration of intervention, and patient characteristics.^4^

The use of 5-Fluorouracil (5-FU) loaded prebiotic-probiotic liposomes has been investigated for improving CRC chemotherapy.^19^ This approach aims to modulate the gut microbiota while delivering chemotherapy, potentially enhancing anti-tumor immune responses and improving treatment outcomes. These liposomes effectively prolong intestinal transport and release of 5-FU, maintaining high drug concentrations at the tumor site while potentially reducing systemic toxicity.^19^ This integration of gut microbiota modulation with conventional cancer therapy represents a promising frontier in personalized medicine approaches to CRC management.

Interventions aimed at modulating the intestinal microbiota may reduce the side effects of chemotherapy and increase the quality of life for these patients.^8^ Given the impact of treatment side effects on adherence and well-being, probiotics warrant further study and potential use in CRC.

## Screening protocol modifications

Screening age is critical given the increasing incidence of early-onset CRC, with studies showing that over 10% of CRC cases occur in individuals younger than 50 years and more than 76.1% of this population have no known risk factors.^20^ Based on current evidence, CRC screening protocols have been lowered to the initial screening age from 50 to 45 years for average-risk individuals.^20^ Simultaneously, preventive strategies should include monitoring of modifiable risk factors, especially dietary components, with significant evidence indicating that higher yogurt consumption is associated with a significant decrease in CRC risk,^8^ and adequate dietary fiber intake provide essential prebiotic substrates that support beneficial gut microbiota and may reduce colorectal neoplasia development.^2^

## Prospects and future directions

Probiotic supplementation is actively being explored as an adjunct to conventional CRC therapies, with studies demonstrating reductions in postoperative infections, enhanced immune responses, and improved efficacy of immunotherapy. Innovative strategies—such as combining probiotics with chemotherapy or employing genetically engineered bacteria to neutralize harmful microbial products-are also under investigation.

Epidemiological studies consistently associate yogurt intake with a reduced risk of CRC; however, conclusive clinical trials remain limited. Addressing this gap would strengthen causal evidence and help establish optimal consumption patterns, probiotic strains, and dosages. Although conducting long-term dietary intervention trials with cancer endpoints is challenging, the use of intermediate biomarkers may offer practical alternatives.

Identifying the bacterial strains most effective for CRC prevention is crucial, as different probiotic species may exert distinct benefits through varied mechanisms. Strain-specific studies are therefore essential for making targeted recommendations and developing specialized probiotic products. Notably, studies have shown that multi-strain probiotic formulations outperform single strains in reducing postoperative infections, suggesting potential synergistic effects in cancer prevention and underscoring the need for further investigation into complex formulations.

Despite these promising findings, large-scale clinical trials are still needed to determine the optimal probiotic strains, dosages, and treatment protocols for CRC prevention and management. Furthermore, addressing racial and ethnic disparities in microbiome composition and CRC outcomes will be critical to ensuring the equitable implementation of microbiome-based interventions.

## Conclusion

The cumulative evidence from epidemiological, experimental, and mechanistic studies supports the hypothesis that probiotic-rich yogurt, particularly formulations containing *Bifidobacterium* and *Lactobacillus* species, exerts a protective role against CRC. This effect is likely mediated through favorable modulation of the gut microbiome, enhancement of immune surveillance, suppression of inflammation, and direct anti-carcinogenic actions. While yogurt and other fermented dairy products are not substitutes for established CRC screening and risk-reduction strategies, their inclusion as part of a balanced, high-fiber diet may offer additional benefits in CRC prevention.

## Authors contribution

Biplab Adhikari: conceptualization, writing – original draft, writing – review & editing, performed all aspects of the work, including critically revising and approving the final version of the manuscript.

## Ethics statement

None.

## Data availability statement

The data utilized in this study can be obtained from the corresponding author upon request.

## Declaration of Generative AI and AI-assisted technologies in the writing process

The authors declare that generative artificial intelligence (AI) and AI assisted technologies were not used in the writing process or any other process during the preparation of this manuscript.

## Funding

None.

## Conflict of interest

The authors confirm that there are no financial interests or personal relationships that could be perceived as having influenced the content of this paper.
